# Supplementary material for: Testing pterosaur ingroup relationships through broader sampling of avemetatarsalian taxa and characters and a range of phylogenetic analysis techniques
Source: PeerJ. 2020 Jul 28;8:e9604. doi: 10.7717/peerj.9604 (PMC7512134; doi:10.7717/peerj.9604)
Supplement: Supplemental Information 2 [file peerj-08-9604-s002.docx]

**Supplementary material - Testing pterosaur ingroup relationships through broader sampling of avemetatarsalian taxa and anatomical characters.**

**1.0 New characters added to the dataset of Britt et al. (2018) and Dalla Vecchia (2019)**

94. Pteroid: 0, absent; 1, present

95. Scapula: 0, distally expanded or parallel sided; 1, tapering to a point

***1.1 Added from the data matrix of Baron et al. 2017a, b***

96. Skull shape: 0, with a deep snout (depth of skull just anterior to the orbit is subequal to depth of the rostral portion of the skull); 1, tapered rostrally (depth of skull just anterior to the orbit is far greater than the depth of the rostral portion of the skull)

97. Transverse groove (sulcus, external mandibular groove) running along the lateral face of the dentary beneath and parallel to tooth row: 0, absent; 1, present.

98. Humerus, proximal articular surface: 0, continuous with the deltopectoral crest; 1, separated by a gap from the deltopectoral crest

99. Dorsal margin of the ilium in lateral view: 0, sinusoidal, or convexo-concave; 1, concave (saddle-shaped), pre and preacetabular and postacetabular processes upturned relative to craniocaudal centre; 2, relatively straight or convex (modified from Baron et al., 2017a)

100. Metatarsal V, length: 0, longer than 50% of metatarsal III; 1, shorter than 50% of metatarsal III

***1.2 Added or modified from the data matrix of Nesbitt 2011, as also modified and updated by Nesbitt et al. 2017***

101. Exoccipital, lateral surface: 0, without subvertical crest (= metotic strut); 1, with clear crest (= metotic strut) lying anterior to both external foramina for hypoglossal nerve (XII); 2, with clear crest (= metotic strut) present anterior to the more posterior external foramina for hypoglossal nerve (XII)

102. Supratemporal fossa: 0, absent anterior to the supratemporal fenestra; 1, present anterior to the supratemporal fenestra

103. Third cervical vertebra, centrum length: 0, subequal to the axis centrum; 1, longer than the axis centrum

104. Ilium, crest dorsal to the supraacetabular crest/rim: 0, absent; 1, present and divides the anterior (preacetabular) process from the posterior (postacetabular) process; 2, confluent with anterior extent of the anterior (preacetabular) process of the ilium

105. Fibula, anterior edge of the proximal portion: 0, rounded; 1, tapers to a point and arched anteromedially

106. Distal end of neural spines of the cervical vertebrae: 0, unexpanded; 1, laterally expanded

107. Trenchant unguals on manual digits I–III: 0, absent; 1, present

108. Ventral margin of the acetabulum: 0, convex; 1, straight; 2, concave ORDERED

109. Perforate acetabulum: 0, absent; 1, present, but semi-perforate; 2, present as fully perforate ORDERED

110. Radius/ulna longer than humerus: 0, present; 1, absent

111. Extensive medial contact between the ischia, but with separated dorsal margins: 0, absent; 1, present

112. Fourth trochanter that forms a sharp flange: 0, absent; 1, present

113. Fourth trochanter asymmetrical, with distal margin forming a steeper angle to the shaft:

114. Cnemial crest that arcs anterolaterally: 0, absent; 1, present

115. Number of premaxillary teeth: 0, four or more; 1, three or fewer

116. Apex of deltopectoral crest situated at a point corresponding to more than 30% down the length of the humerus: 0, absent; 1, present

***1.3 Other additional characters***

117. Lacrimal process of the jugal: 0, inclined anteriorly; 1, perpendicular to the dental plane; 2, reclined posteriorly (modified from Vidovic and Martill, 2014)

118. External naris: 0, small, height similar to or greater than anteroposterior length; 1, elongated; 2, very tall and elongated (modified from Lü et al., 2009)

119. Neural spines and hypapophyses of caudals: 0, slender and rod-like; 1, robust

120. Pubis and ischium fusion at midshaft: 0, completely fused; 1, only partially in contact and fused; 2, not fused

**2.0 Trees recovered in the analyses**

**
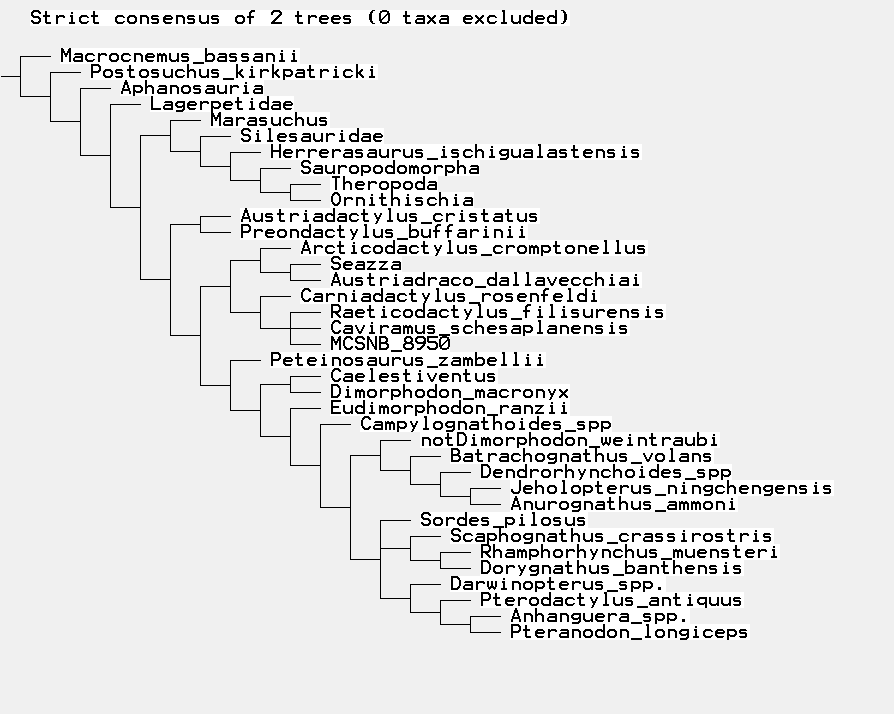
**

**Figure Sup. Inf. 1. Raw output of TNT analysis that used the modified data of Dalla Vecchia (2019).**

**
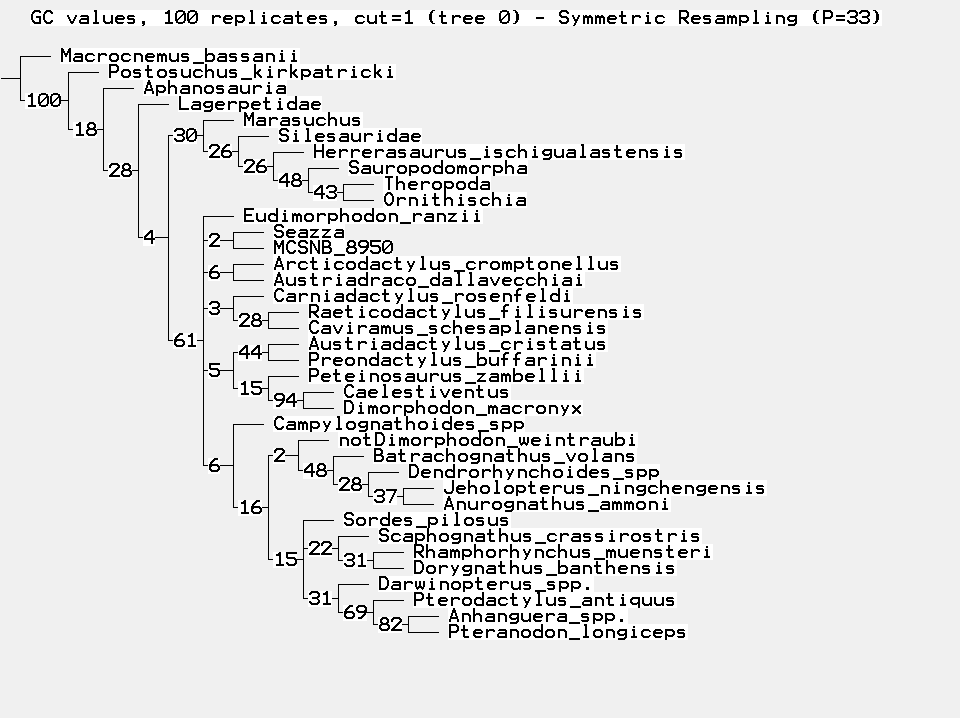
**

**Fig. Sup. Inf. 2. Trees recovered after TB branch swapping resampling, following the protocol of Ezcurra (2016), with all taxa included.**

**
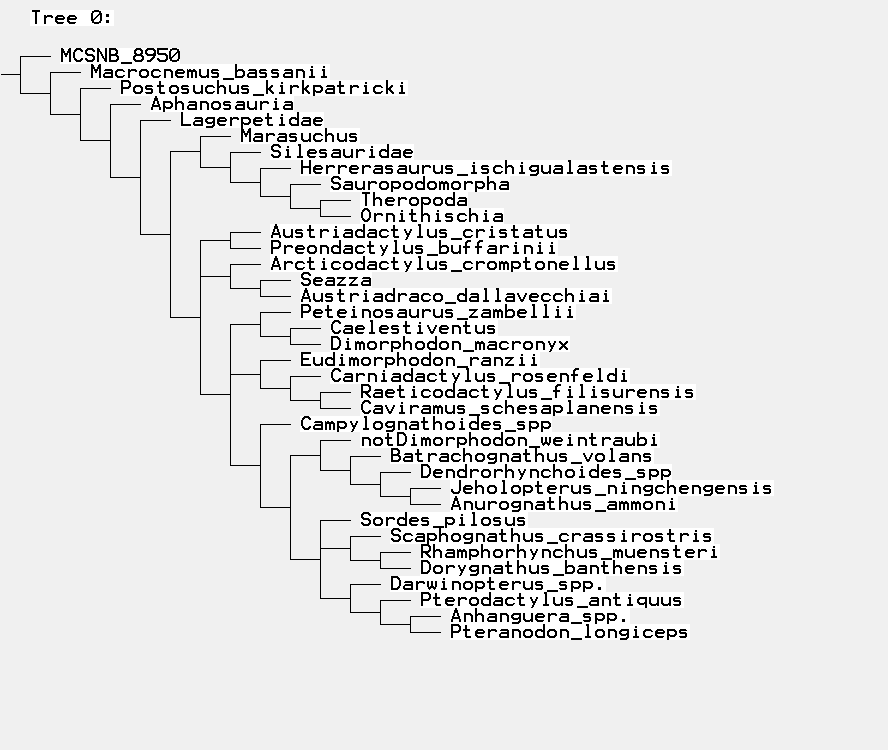
**

**Fig. Sup. Inf. 3. Reduced strict consensus produced in analysis two, following the exclusion of the wildcard taxon MCSNB 8950, as was also done in the analysis of Britt et al. (2018).**

**
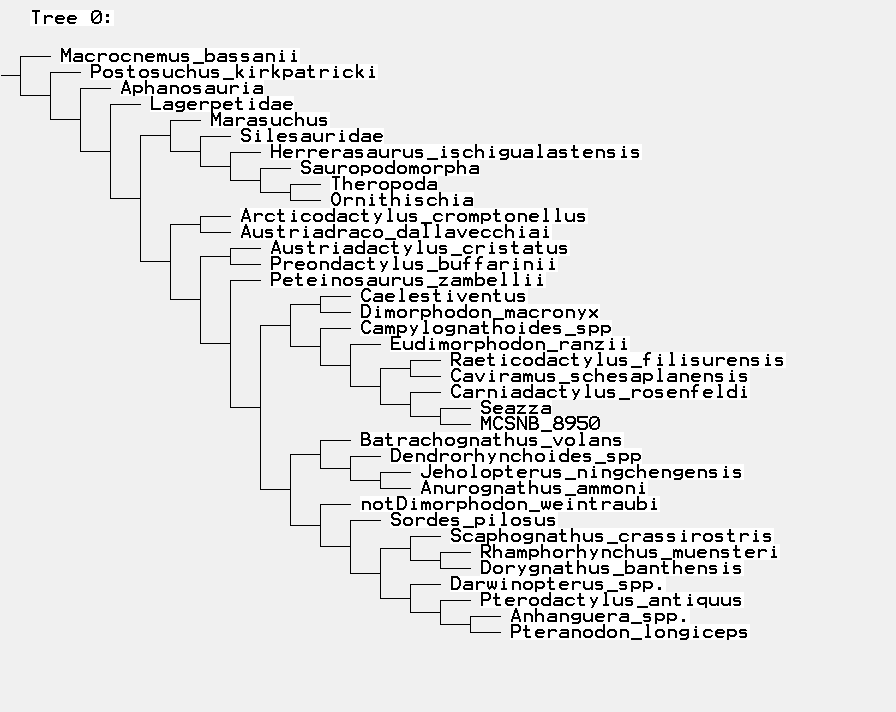
**

**Fig. Sup. Inf. 4. Single most parsimonious tree produced when using implied weights implementation of parsimony with k = 3.**

**
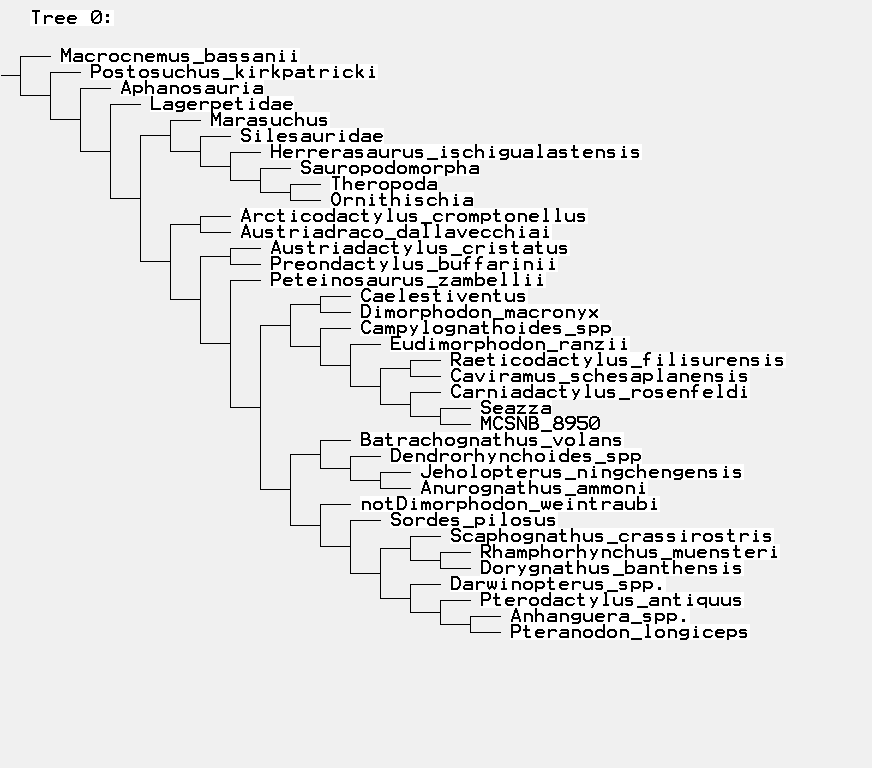
**

**Fig. Sup. Inf. 5. Single most parsimonious tree produced when using implied weights implementation of parsimony with k = 5.**

**
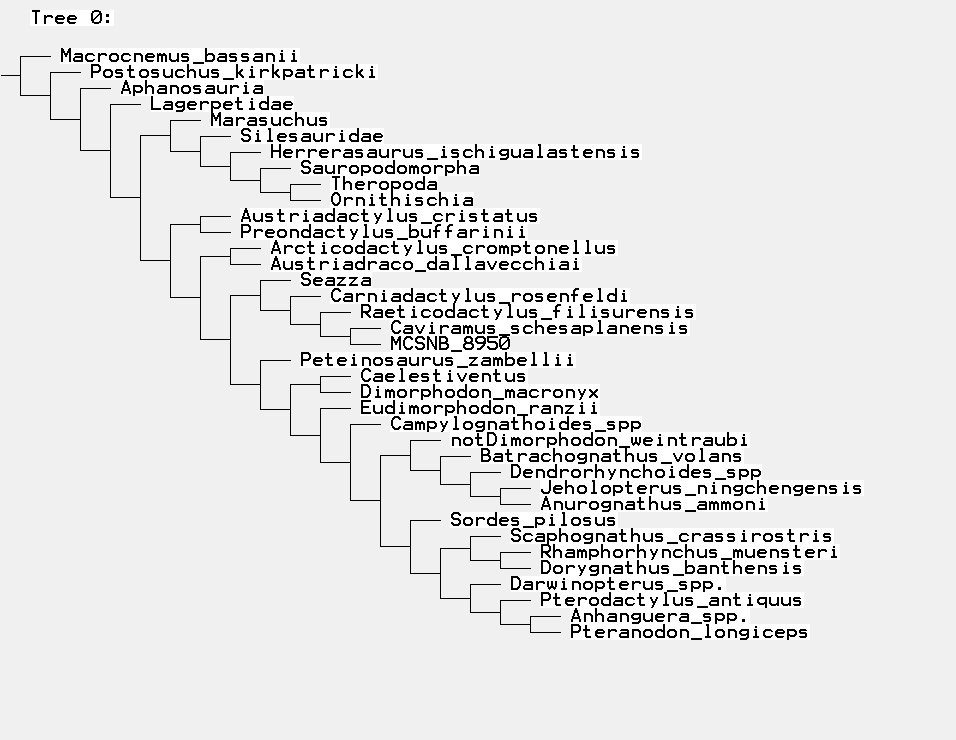
**

**Fig. Sup. Inf. 6. Single most parsimonious tree produced when using implied weights implementation of parsimony with k = 10 and more.**

**Nomenclatural acts.**

**LSID:** urn:lsid:zoobank.org:pub:BE350658-1D5C-456B-B129-FFDE827E7DDF
